# New Exploration of Chinese Herbal Medicines in Hepatology

**DOI:** 10.1155/2016/3056438

**Published:** 2016-07-20

**Authors:** Yibin Feng, Man-Fung Yuen, Qihe Xu, Xiao-Yan Wen, David Q. H. Wang

**Affiliations:** ^1^School of Chinese Medicine, Li Ka Shing Faculty of Medicine, The University of Hong Kong, 10 Sassoon Road, Pokfulam, Hong Kong; ^2^Division of Gastroenterology and Hepatology, Queen Mary Hospital and Department of Medicine, Li Ka Shing Faculty of Medicine, The University of Hong Kong, Pokfulam, Hong Kong; ^3^Centre for Integrative Chinese Medicine and Department of Renal Sciences, Division of Transplantation Immunology and Mucosal Biology, MRC Centre for Transplantation, Faculty of Life Sciences & Medicine, King's College London, London WC2R 2LS, UK; ^4^Zebrafish Centre for Advanced Drug Discovery, Keenan Research Centre of Biomedical Science, Li Ka Shing Knowledge Institute, St. Michael's Hospital & Department of Medicine, Physiology, Faculty of Medicine, University of Toronto, Toronto, ON, Canada M5S 1A8; ^5^Division of Gastroenterology and Hepatology, Department of Internal Medicine, Edward Doisy Research Center, Saint Louis University School of Medicine, St. Louis, MO 63104, USA

## 1. Objectives of This Special Issue

Chronic liver diseases (CLD) are serious health-threatening problems worldwide. In particular, it is a significant healthcare burden in Southeast Asian countries where viral hepatitis is common. Regardless how the diseases are initiated, without proper intervention, CLD may gradually progress towards end-stage liver disease (ESLD), for which liver transplantation remains the only solution. Although this progression may be slow and gradual, there is no evidence showing that it could be reversed when cirrhosis has developed. Preventive intervention that may impede the progression of CLD is clinically favourable. Management of CLD depends on the etiology, and supporting treatment and hepatoprotective medicines are often provided. However, drug resistance and various adverse effects can lead to failure of treatment.

Many countries have their own traditional medicines and herbal medicines are the main therapeutic materials in most traditional medicines. In the past decades, herbal medicines have been widely used to treat liver diseases. The pros and cons of herbal medicines for treatment of liver diseases are intensively debated in recent years. The main goal of this special issue is to showcase the latest research in this research area to promote academic exchanges and disseminate the state of the art.

## 2. Analysis of This Special Issue

This special issue covers cutting-edge research topics on herbal medicines in hepatology, including original research articles and current reviews in the following categories:

- Basic, translational, and clinical studies of herbal medicines for preventing and treating liver diseases and their complications.
- Liver injury induced by herbal medicines and herb-drug interaction.
- Systematic reviews on studies of herbal compounds, herbs, and herbal formulae for liver diseases.

After a rigorous peer-review process, the special issue accepted nine manuscripts, including six original research papers and three review papers, out of 30 submissions. The accepted papers were financially supported by various funding schemes from different countries and regions including Mainland China, Hong Kong, Canada, Egypt, and South Korea.

Original research articles in this special issue focus on either herbal medicine compounds or composite herbal extracts, reporting novel pharmacological activities and applying new research methodology. The study from Ghareeb and colleagues investigated the therapeutic potential of berberine against neurotoxicity-related Alzheimer's disease, a secondary complication of nonalcoholic steatohepatitis (NASH) in a rodent model of NASH, and demonstrated that berberine had anti-inflammatory and antioxidative activities and selectively suppressed AChE and HMG-CoA. Interestingly, study from Wang and colleagues also reported that berberine regulated viability and activation of hepatic stellate cells and prevented fibrogenesis in* in vitro* and* in vivo* liver disease models. The two papers highlighted the potential of berberine in the treatment of CLD and related complications.

Kim and colleagues reported a herbal formula used for antifibrotic treatment in Korean Medicine. This formula, known as CGXII, effectively prevented dimethylnitrosamine-induced hepatic fibrosis. Chen and colleagues reported the preventive effect of Buzui recipe, a Chinese Medicine recipe, on alcoholic intoxication in mice and the effects may be associated with the antioxidative activity of the recipe. Wang and colleagues offered new insights into the research methodologies on pharmacological activities of Chinese Herbal Medicine. With untargeted metabolomics combined with bioinformatic approaches, the team unveiled potential mechanisms underlying the therapeutic effect of turmeric extract in treating nonalcoholic fatty liver diseases (NAFLD).

Highlighted in this special issue also is two meta-analysis papers on the efficacy and safety of therapies combining Chinese Medicine and conventional treatments as follows. He and colleagues reported on the clinical effects of oxymatrine, a compound isolated from Sophorae Flavescentis Radix, on lamivudine-induced tyrosine-methionine-aspartate-aspartate (YMDD) mutation, a common mutation that causes serious drug resistance in patients with HBV infection. By recruiting data from 324 clinical studies, the authors confirmed that YMDD mutation was much lower in patients receiving both oxymatrine and lamivudine. Another meta-analysis study focused on the efficacy and safety of a combined therapy of arsenic trioxide and transcatheter hepatic arterial chemoembolization (TACE) in treating primary hepatic carcinoma. The study recruited data from 15 randomized controlled trials (RCTs). Comparative analysis on 530 cases of treatment with 525 control cases revealed that the combined treatment appeared effective and safe in general. However, both studies await further clarification by future large-scale, high-quality clinical trials.

Animal models play important roles in evaluating herbal medicines for the treatment of liver diseases. Experimental evaluation of the pharmacological effects of herbal medicines on liver diseases is often conducted in various animal models. A review by Tan and colleagues comprehensively summarized the animal models of liver diseases treated by herbal medicines in recent years. The authors concluded that a great challenge remained to establish a perfect preclinical evaluation and suggested that several models in combination may be better in achieving clinical-relevant outcomes. Furthermore, the authors highlighted the emerging zebrafish model as a novel approach in evaluating the efficacy of herbal medicines for liver diseases.

As a feature, bear bile, one of the animal parts from bears, is an important constituent in liver-protecting prescriptions in Chinese Medicine, but it incurs controversy due to its animal origin. The commentary by Li and colleagues identified several potential herbal substitutes including Coptidis Rhizoma, which were shown to be able to replace bear bile in terms of its pharmacological effects as well as clinical efficacy in treating liver diseases.

In summary, this special issue has collected state-of-the-art reports on herbal treatment of liver diseases and explored new heights in hepatology. Although it does not cover all aspects of progress in this area, we hope that it will be of interest to researchers in the field of ethnopharmacology and life sciences, as well as clinical hepatology, and will lay the foundation for future development.

## 3. The Road Ahead

**Figure 1:**
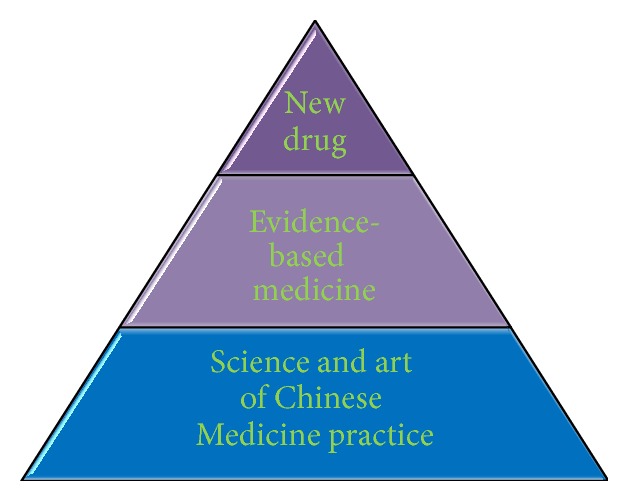
Three categories of research in Chinese Medicine. In the long term, we hope to establish a research platform on Chinese Medicines by interdisciplinary technologies and concepts in molecular medicine, omics, informatics, nanotechnology, systems biology, integrative medicine, individual medicine, translational medicine, evidence-based medicine, and so forth. We propose a research pyramid platform by the following three categories: (a) research on new drug discovery from Chinese Medicines, (b) research on evidence-based complementary and alternative medicine from Chinese Medicine, and (c) research on the science and art of Chinese Medicine and other therapeutic formalities in Chinese Medicine practice.

About a hundred years ago, Chinese Medicine was the only medical system in China and it has been merged into the mainstream medical system nowadays in Mainland China. It is also one of the officially recognised medical systems in Taiwan, Macau, and Hong Kong as well as several Asian-Pacific and European countries. Based on two thousand years of clinical experiences and over a hundred years of scientific research, Chinese Herbal Medicine has now become an important resource for drug discovery as well as a valuable complementary and alternative medicine worldwide. The Chinese Medicine-inspired discovery of antimalarial drug artemisinin by Youyou Tu was honoured by a Lasker Award in Clinical Medicine in 2011 and a Nobel Prize in Physiology or Medicine in 2015, and this has impelled Chinese Medicine research. Indeed, discovery of drug candidates for liver diseases has been inspired by Chinese Medicine too. For example, based on ethnopharmacological properties of Chinese Medicine, many studies on bear bile and other animal bile have identified its chemical profiles, pharmacological effects, and mechanisms of action; in particular, ursodeoxycholic acid (UDCA) isolated from bear bile has been synthesized and developed to be a drug for treating (cholesterol) gallstones [1] and its derivatives ursodiol is the only FDA approved drug to treat primary biliary cirrhosis [2]. Looking forward, what more can we do with Chinese Herbal Medicine? The high-throughput approach in discovering active compounds from Chinese Medicines for liver disease treatment remains very attractive, and gaining further clues and hints through continued exploration of clinical experiences and traditional wisdoms should also be appreciated. From 2014 to 2015, a supplement series in Science have been published to illustrate a panorama of the art and science of Traditional Medicine [3–5]. In one of the* Science *supplements, two editors of this special issue, Xu et al. have critically discussed the hunt for antifibrotics and profibrotics from botanicals and proposed an efficacy-based strategy for discovery of botanical antifibrotics [6]. Herein, we propose a research pyramid (Figure 1), which links traditional practice, evidence-based medicine to the discovery of new drugs.

Both single pure compound and multiple components are important for scientific research for Chinese Medicine. However, apart from purified compounds or individual herbal extracts, composite formulae are the most commonly used in the clinical practice of Chinese Medicine. Given their nature, it is still difficult to produce herb-derived natural products with identical quality from laboratory to laboratory or even batch to batch in the same laboratory, as the different resources or minor variance in herb growth conditions and production protocols would largely harm reproducibility. The key point is to assure stable research materials and follow good practices (GAP, GMP, GLP, and GCP) to facilitate reliable and reproducible results. Guidelines of good practice in handling herbal materials from farm to dispensary may be available at national level but vary across countries and region and lack at global level. Some pioneering attempts have been made since 2009 to develop and disseminate good practice guidelines on Chinese Medicine research [7, 8]. Further development of such guidelines would heavily rely on international, intersectoral, and interdisciplinary collaborations.

Another challenge in the current research on traditional medicine is the uncertainty of mechanisms of action and the possibility of multiple targets. Containing multiple components, herbal medicine may act on different targets to regulate biological responses in cellular, animal, and human systems. This polypharmacological property of herbal medicine requires data capturing and mining at multiple layers of biological responses necessary for systematic understanding of its action. For this, an omics- and systems biology-based approach will be needed. Omics are powerful technologies for exploring characteristics of herbs in its nature of systems biology. By using systems biology and omics technologies, omic data can be tracked and understood by using available online database. Construction of intralayer and interlayer regulatory network gives rise to multidimensional pictures connecting the chemical and biological identity of herbal medicine.

Like any other traditional medicines and ethnomedicines, Chinese Medicine relies on natural resource and some species (plants or animals) effective for liver disease are endangered. The medicinal use of these endangered species has raised ethical and legal concerns. Identification and synthesis of bioactive components from endangered species and search for replacement for endangered species should thus be an essential topic in current and future research.

**Figure 2:**
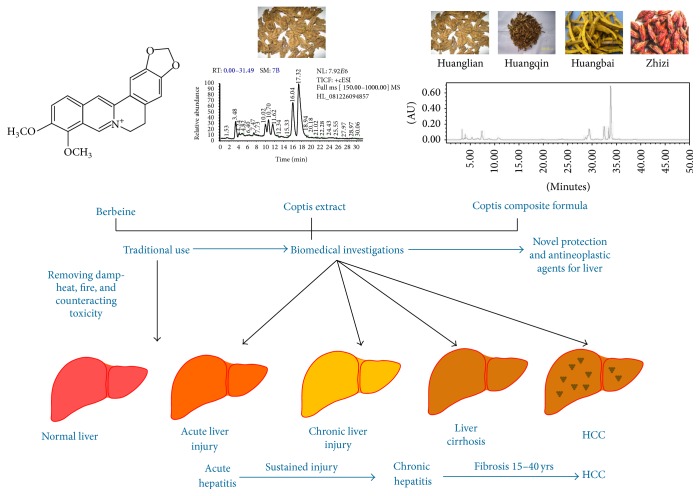
Research strategy proposed for Chinese Herbal Medicines in hepatology: Coptis (huanglian in Chinese) and its single pure compound and composite formula used as an examples. Using different materials, disease models, and omics data, research will be clarified for bioactivity relevance of the chemical profile, pharmacological network of bioactive-relevant compounds, and interactions amongst multiple components on molecular targets in this complicated system of Chinese Medicine.

Of note, three papers in this special issue focused on the pharmacological properties of berberine, a compound isolated from a herb commonly used in Chinese Medicine, including for the treatment of liver diseases. Berberine is an active natural compound that possesses multiple pharmacological properties, and the use of berberine and berberine-containing herbs in treating liver diseases has been systemically reported by the Lead Guest Editor's laboratory [9–13], including for the treatment of acute [9] and chronic liver damage [10] and hepatic cancer [11–13]. Thus, berberine and berberine-containing herbs have the potential to be developed into novel treatments for liver diseases and these studies can be a paradigm for study of Chinese Herbal Medicines in hepatology (Figure 2).

Finally, efficacy, safety, and mechanisms of action remain obscure for most Chinese Medicines widely used in the treatment of liver diseases. Upon publication of this special issue, a new exploration of Chinese Herbal Medicines in hepatology begins.
